# Microbial community shift on artificial biological reef structures (ABRs) deployed in the South China Sea

**DOI:** 10.1038/s41598-023-29359-5

**Published:** 2023-03-01

**Authors:** Hala F. Mohamed, Amro Abd-Elgawad, Rongshuo Cai, Zhaohe Luo, Lulu Pie, Changan Xu

**Affiliations:** 1 Third Institute of Oceanography, Ministry of Natural Resources, Xiamen, 361005 People’s Republic of China; 2 Botany & Microbiology Department, (Girls Branch), Faculty of Science, Al-Azhar University, Cairo, Egypt; 3 Tourism Developing Authority, Central Administration for Environmental Affairs, Cairo, Egypt

**Keywords:** Microbial ecology, Ocean sciences

## Abstract

Many Artificial Reefs (ARs) have been used worldwide for marine habitat and coral reef restoration. However, the microbial community structure that colonize the ARs and their progressive development have been seldom investigated. In this study, the successive development of the microbial communities on environmentally friendly Artificial Biological Reef structures (ABRs)^R^ made of special concrete supported with bioactive materials collected from marine algal sources were studied. Three seasons (spring, summer and autumn), three coral reef localities and control models (SCE) without bioactive material and (NCE) made of normal cement were compared. The structure of the microbial pattern exhibited successive shifts from the natural environment to the ABRs supported with bioactive materials (ABAM). Cyanobacteria, Proteobacteria, and Planctomycetota were shown to be the most three dominant phyla. Their relative abundances pointedly increased on ABAM and SCE models compared to the environment. Amplicon Sequence Variant (ASV) Richness and Shannon index were obviously higher on ABAM models and showed significant positive relationship with that of macrobenthos than those on the controls and the natural reef (XR). Our results offer successful establishment of healthy microbial films on the ABR surfaces enhanced the restoration of macrobenthic community in the damaged coral reefs which better understands the ecological role of the ABRs.

## Introduction

Over the past decade, many underwater artificial structures have been used worldwide to resist coral decline as well as repairing of marine habitats damaged by environmental disturbances including human impact and climate change^1–4^. Different substances have been used to construct ARs, for example, cement, plastic, metal, rock, wood, fiberglass, and clay^2,3,5^.

Artificial reefs are concerned with restoring the typical habitat by gradual settlement of periphyton (microorganisms, algae and invertebrates) on their surfaces then succession stages follow^5^, in which four main phases are recognized: (1) instantly following installation, exterior acclimatizing takes place by absorption of organic and inorganic particles with the formation of a prefilm (seconds to minutes); (2) primary adherence and development of prokaryotic cells as well as lower eukaryotes on the substratum or to the primary formed film (minutes to hours); (3) germination of algal spores in addition to invertebrate larvae (days to weeks); and (4) establishment of a multi-community of advanced and variable species on the substrates (weeks to years)^6,7^. More attention has been drawn towards invertebrate and macroalgal communities colonizing ARs with little focus on the microbial community pattern although they are the very first developer of biofilms on Ars. However, they got disturbed by biological and nonbiological constraints. To our knowledge, only few studies have examined the microbial community composition and succession on ARs^8,9^. Thus, exploring the development of microbial communities over time enhances revealing the ecological part of ARs^10^. Microbial community established on a solid substratum rise in abundance over time at the primary phase of biofilm establishment^11–13^. This is in consistence with Abed et al. who reported a substantial reduction in the bacterial α-diversity over the 28 days of installing Ar models^14^.

In aquatic networks, microbial biofilms are the initial manufacturers that provide the biochemical needs and support the settlement and recruitment of eukaryotic larvae and algal spores^6,15^. Physical, biological and chemical factors govern the formation, development and succession of microbial biofilm^16^. Although attachment and development of microbial community structure have been proven to be shaped according to the substrate shape, color, roughness, material^6^, but also seems to rely more on geographical distances^17^ in addition to environmental biochemical parameters^18^. Microbiota is influenced by the AR materials to as far as the species abundance and richness levels^19,20^. Close tracing for the emergence of such microbial communities over time in coincidence with the recovery of benthic habitat associated with artificial substrates could be the puzzle solving key and the chain link to the coral reef ecosystem rehabilitation. In Weizhou Island, the biggest and youngest volcanic island in China sea, many ARs have been applied to understand why it could not benefit from the climate change and the Sea Surface Temperature (SST) rise according to the refuge theory to resist coral decline with a high latitude environment. However, the microbial community in coral reefs remains unexplored specially in correlation with the two types of impact and to the used artificial structures. In this study, microbial communities covered artificial biological reefs (ABRs)^R^ for sexual marine habitat restoration (patency registration No. 202111290154.5, unpublished data) of affected sites were investigated in comparison to the natural coral ecosystem of three selected sites in Weizhou Island. Moreover, correlation between microbial and macrobenthic diversity succession on the ABRs is explored over three seasons. (ABRs) are made up of special concrete structures supported with specific bioactive materials of marine algal origin known to support marine habitat larval attraction including corals (patency registration No. 202111290154.5, unpublished data). The three selected sites in Weizhou Island were chosen according to the health status of the coral reef and according to live and dead coral coverage.

## Results

### Microbiome assemblages and comparisons of natural reef substrate (XR) and ABRs at high and low taxonomic levels

After filtering unqualified sequences, for the natural reef samples (XR group; As, Bs, and Cs) collected from the three sites A, B, and C, a total of 40,491, 39,986, and 30,765 clean 16S tags were obtained in March, 30,769, 36,272, and 28,316 in July and 25,227, 31,466, and 33,803 in October respectively. Archaea showed a very low percentage (< 0.5%) of combined Bacteria and Archaea. We only focus on the bacteria in the following analysis. XR samples, yielded 497, 194, and 356 unique ASVs in March, 489, 37, and 372 in July and 73, 89, and 85 in October, representing five bacterial phyla (based on SILVA classification), with a maximum of 497 unique ASVs in sample As in March (Table 1).

**Table 1:** Co-occurrence network characteristics calculated for the microbial genera on the three treatments plus XR samples.

Samples_name	Modulatory	Average degree	Average network distance	Density
ABAM	0.219	6.455	7	0.307
SCE	0.572	3.4	5	0.179
NCE	0.28	4.435	8	0.202
XR	0.29	3.375	5	0.225

Nine samples were collected per season from the surface of the ABRs with different treatments installed in the three sites (Table 2). Three samples from each of ABAM, SCE, and NCE groups. The total of clean 16S tags obtained in the three seasons for all samples is shown in Supplementary Table S1. ABAM group; supported with bioactive materials, includes samples A10, B25, and C40 collected from site A, B, and C respectively. They yielded 356, 419, and 418 unique ASVs in March, 288, 486, and 376 in July and 535, 412 and 488 in October, representing five bacterial phyla, with a maximum of 535 unique ASVs in sample A10 in October (Supplementary Table S1). SCE Group includes samples; A20, B34, and C55 representing microbial films from the control ABR model made of specific cement with no supplement, yielding 380, 419, and 438 unique ASVs in March, 144, 272, and 329 in July and 625, 451, and 323 in October, representing five bacterial phyla, with a maximum of 625 unique ASVs in sample A20 in October (Supplementary Table S1). While NCE Group represented by samples; A59, B36, and C58 collected from the microbial films grown on the negative control model made of normal cement yielded 160, 337, and 415 unique ASVs in March, 202, 257 and 555 in July and 208, 290, and 368 in October, representing five bacterial phyla (based on SILVA classification), with a maximum of 555 unique ASVs in sample C58 in July (Supplementary Table S1).

**Table 2:** Samples collected from the surface of the ABRs with the three different treatments and the natural reef sediment (XR). ABAM indicates the ABR model made of specific cement supported with bioactive materials, SCE indicate the control ABR model made of specific cement with no supplement, NCE indicates the negative control model made of normal cement with no added bioactive materials, and XR represent the natural reef samples (As, Bs, Cs).

ABR treatment	Model No/Site A	Model No/Site B	Model No/Site C
ABAM	A10_Mar/A10_Jul/A10_Oct	B25_Mar/B25_Jul/B25_Oct	C40_Mar/C40_Jul/C40_Oct
SCE	A20_Mar/A20_Jul/A20_Oct	B34_Mar/B34_Jul/B34_Oct	C55_Mar/C55_Jul/C55_Oct
NCE	A59_Mar/A59_Jul/A59_Oct	B36_Mar/B36_Jul/B36_Oct	C58_Mar/C58_Jul/C58_Oct
XR (Natural reef)	As_Mar/As_Jul/As_Oct	Bs_Mar/Bs_Jul/Bs_Oct	Cs_Mar/Cs_Jul/Cs_Oct

### Temporal succession of bacterial community

**Figure 1:**
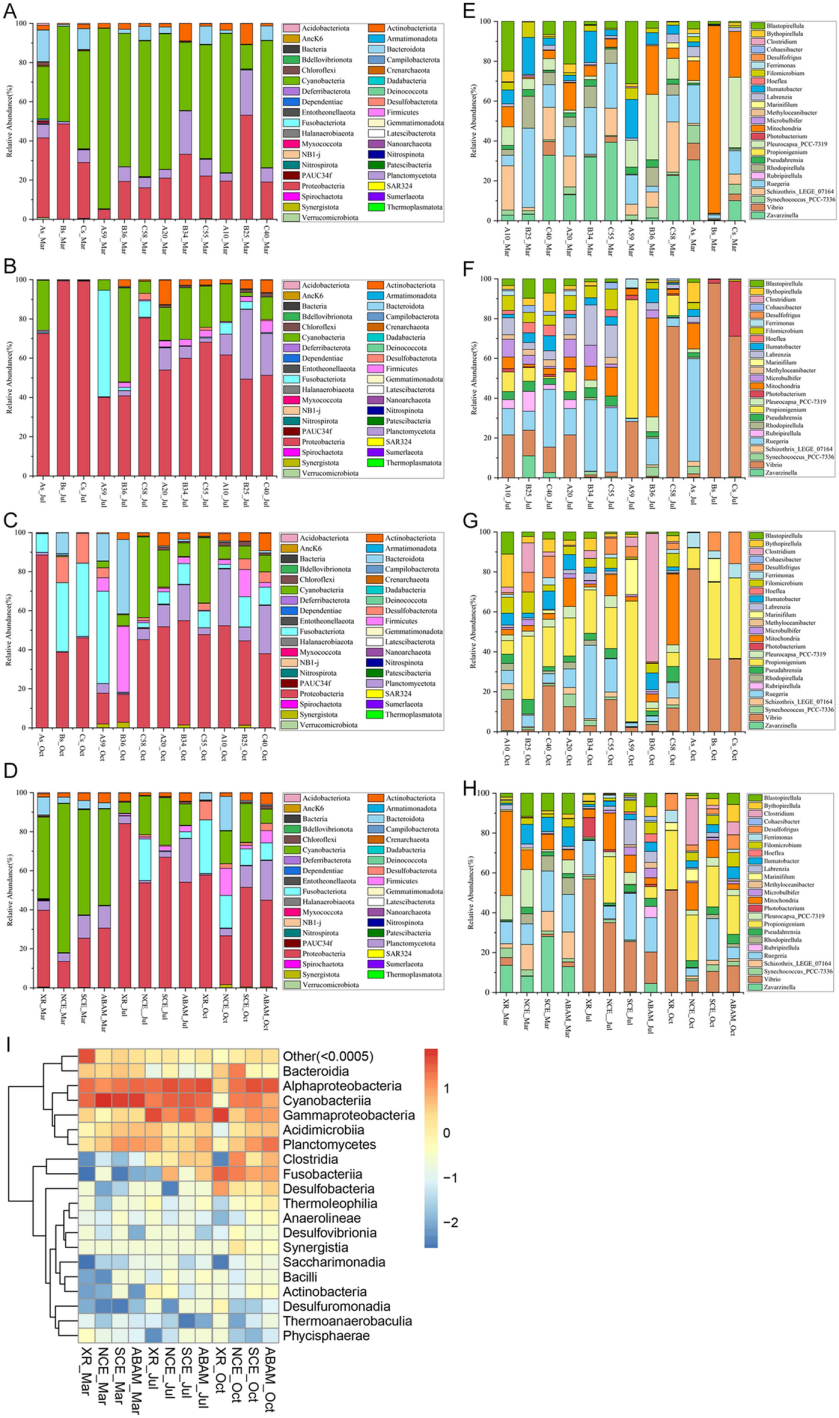
Temporal dynamics of the relative abundances of top bacterial phyla and genera of the three sampling treatments (ABAM, SCE and NCE) Plus the samples from the natural environment (XR) for the three different localities. (**A**–**C**) Relative abundance of key bacterial Phyla in March, July, and October respectively. (**D**) Relative abundance of key bacterial Phyla of total bacteria grouped for all three localities with the different treatments plus the XR. (**E**–**G**) Relative abundance of dominant bacterial genera in March, July, and October respectively. (**H**) Relative abundance of key bacterial Genera of total bacteria grouped for all three localities with the different treatments plus the XR (**I**) Heat map of the relative abundance of the 20 most abundant classes over time.

Microbiome assemblages of XR and microbial films from ABRs surfaces displayed quite different profiles among different locations and seasons at all levels, phylum and genus levels are shown (Fig. 1).

During March, the phylum Proteobacteria and Cyanobacteria equally dominated the natural reef microbiome assemblages with a relative abundance of about 53%. While all ABRs microbiome were dominated by Cyanobacteria followed by Proteobacteria and Planctomycetota with the NCE samples exhibiting the highest Cyanobacteria abundance reaching 76%, indicating that all models could attract Cyanobacteria, Proteobacteria and Planctomycetota (Fig. 1A). Expectedly, in July diversity started to increase in the ABR samples with the highest detected in the ABAM treatments compared to the XR samples which showed the lowest diversity specially in site B and C with full abundance of Proteobacteria. Proteobacteria started to overgrow the Cyanobacteria with all samples with slightly different relative abundance. In addition, sample A59 which corresponds to negative control NCE from site A showed significant appearance and abundance of Fusobacteriota. Also, Planctomycetota presented its highest level of abundance with the ABAM treatments from the three sites (Fig. 1B). In October, bacterial diversity reached the highest level among all seasons (Fig. 1C).

Regardless of the bacterial relative abundance differences among different sites, for a complete picture on the efficiency of ABAM treatment in restoring a healthy biofilm, a combined Phyla picture of all three sites at separate treatment over the three months is analyzed (Fig. 1D). It was clearly noticed that Proteobacteria over grew the Cyanobacteria in all the samples and reached its maximum relative abundance in July season (66.28%, 54.68%, 58.02%, 51.30% with XR, NCE, SCE and ABAM respectively). In addition, other phyla started to appear in all samples and increased in abundance in the ABAM treatment as well as the SCE treatment, such as Fusobacteriota, Planctomycetota and Firmicutes. Interestingly, Proteobacteria started to decline in October with an increase in the diversity with the treatment samples, while XR group lost the Firmicutes and Cyanobacteria phyla and developed a significant abundance of Desulfobacterota (10.40%) and Fusobacteriota (28.96%). All treatment samples continued to decrease the abundance of Cyanobacteria with ABAM reached the minimum relative abundance (7.20%), highest Proteobacteria, Chloroflexi, and Actinobacteria with (44.26%, 1.81% and 4.33% respectively). It’s worth mentioning that among the different treatments, ABAM showed the lowest abundance of Fusobacteriota (8.54%) and Cyanobacteria (7.20%) but showed the highest abundance of Actinobacteriota (5.79%), Desulfobacteriota (3.69%) and Chloroflexi (1.81%). In general, Phylum analysis indicates that Proteobacteria replaced Cyanobacteria as the dominant clade as time going. Location wise, site B exhibited the highest level of abundance of Proteobacteria in March and July, highest Plactomycetota and Firmicutes in October.

On the Genus-level, variations in diversity as well as relative abundance among bacterial genera were more obvious than on the Phylum level site/treatment wise. In March, XR samples exhibited variations in the genus abundance among different sites, where site B and C showed very low diversity. Site A was most dominated with Zavarzinella (50.16%), site B with Mitochondria (94.78%), and site C with Pleurocapsa_PCC-7319 (30.10%). On the other hand, treatment models started to attract more diversity in the three sites. ABAM treatment showed variation in the dominant species among different sites, for example, site A exhibited significant abundance of *Schizothrix* and *Blastopirellula* while site B showed significant abundance of *Rugeria*, *Illumatobacter* and *Rhodopirellula*. However, SCE treatment showed more consistent results with the three sites but with relatively different abundance level. NCE showed less diversity than ABAM and SCE treatments but more than that of the XR group (Fig. 1E). In July, diversity has increased significantly with the ABAM treatment in all sites compared to the rest of treatments and the XR group. In addition, *Vibrio* Genus appeared dominantly in the XR group and in the NCE samples from site C and A but not B (Fig. 1F). In October, *Propiogenium* showed significant abundances in all samples except for B36 which showed significant abundance of *Clostridium*. *Propiogenium* shared equally the abundance with *Vibrio* in Bs and Cs samples. In general, pattern of ABAM and SCE looked more consistent in all sites with slight fluctuations in abundances, in contrast to NCE samples with the dominance of *Propiogenium*, *Clostridium* and Mitochondria in A59, B36 and C58 respectively (Fig. 1G). In the combined genera figure for each treatment (Fig. 1H), the XR group presented the highest relative abundance of *Vibrio* and *Propionigenium*, which developed in July and increased in August. In contrast to the treatments models which showed significant decrease of *Vibrio* from July to October. On the other hand, diversity increased significantly over time with the ABAM treatment models compared to the XR groups (Fig. 1H).

The variation in community pattern among different treatment samples using a heat map of the relative abundance of the 20 most abundant Class displayed average linkage among groups over the three seasons (Fig. 1I). The figure shows that ABAM treatment started to develop Alphaproteobacteria, Planctomycetes and Cyanobacteria in March, and that while the first two increased over time until reached the maximum in October, Cyanobacteria exhibited significant decrease.

**Figure 2:**
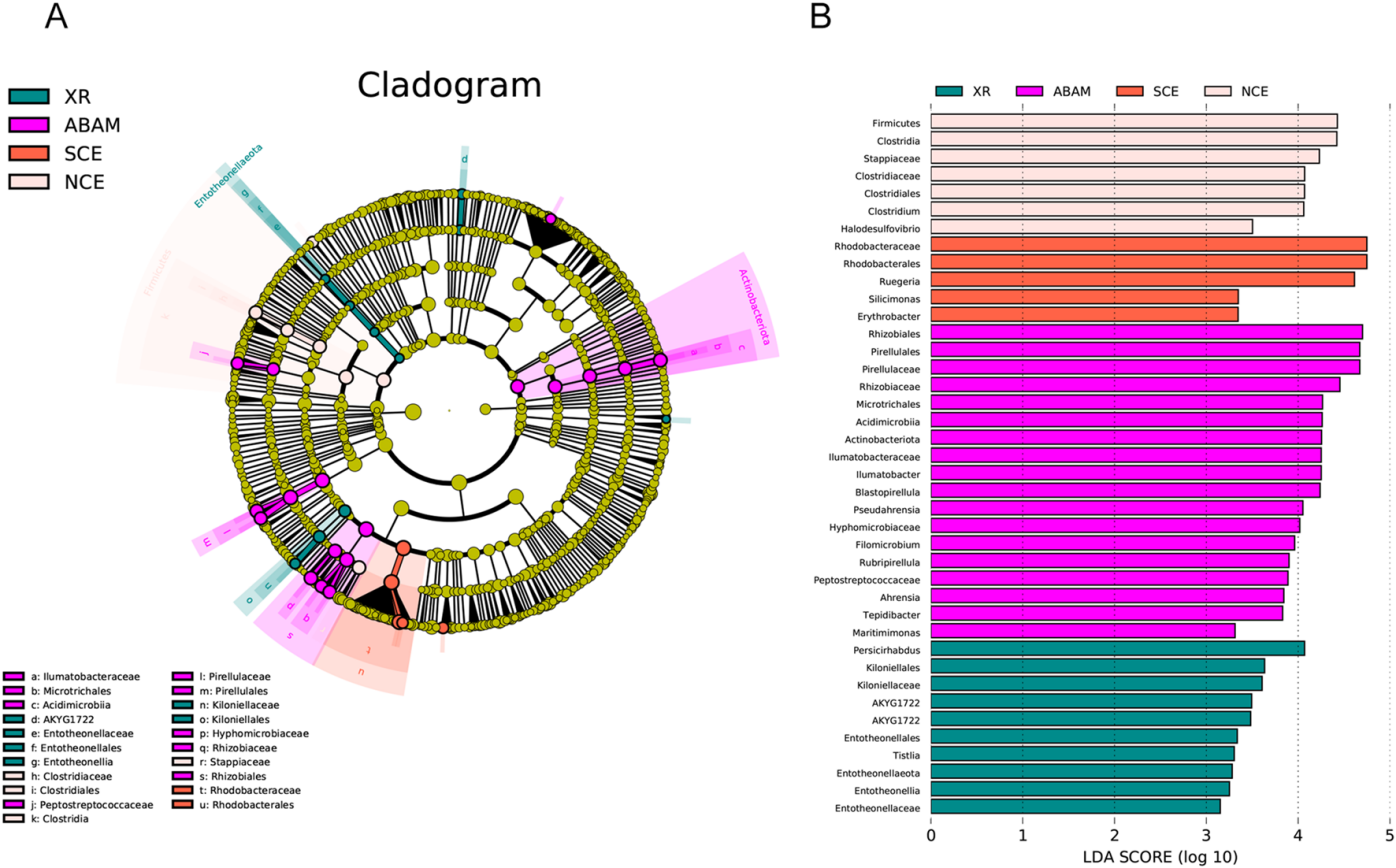
(**A**) Lefse clustering tree. Different colors represent different groups over the three seasons. The yellow nodes represent microbial taxa without significant differences among groups. Nodes with different colors represent the microbiota that plays an important role in the group represented by the color. A color circle represents a biomarker, and the legend in the upper right corner is the name of biomarker. From the inside to the outside, each circle is the taxa at the level of phylum, class, order, family, and genus. (**B**) Histogram showing the distribution of Least Discriminant Analysis (LDA).

Moreover, the temporal and substrate influence on the microbial biofilm on the ABAM, SCE, NCE and the XR samples, were studied using LEfSe analysis (Fig. 2A). The obtained result is illustrated by a Cladogram in green, purple, red and cream nodes, which represent microbial taxa that had a fundamental role in the XR, ABAM, SCE and NCE groups respectively. Illumatobacteraceae, Microtrichales, Acidimicrobiia, Peptostreptococcaceae, Pirellulaceae, Pirellulales, Hyphomicrobiaceae, Rhizobiaceae and Rhizobiales are shown to have important role in ABAM treatment**.** In addition, LDA was used to detect variations in taxon pattern among the four samples (Fig. 2B). Different colors represent the significantly different taxon with LDA value higher than the preset value, that is, biomarker with statistical variation. The original preset value is 2.0 (only the value of LDA higher than 2 will be indicated in the figure). The color of the histogram indicates their respective groups, and the length indicates LDA score, that is, the impact of species with clear differences among different groups. LDA identified 10, 18, 5, and 7 biomarkers with XR, ABAM, SCE, and NCE respectively (Fig. 2B), which reflects that the dominant species of the microbial communities varied significantly over time and substrate.

### Alpha and beta diversity

**Figure 3:**
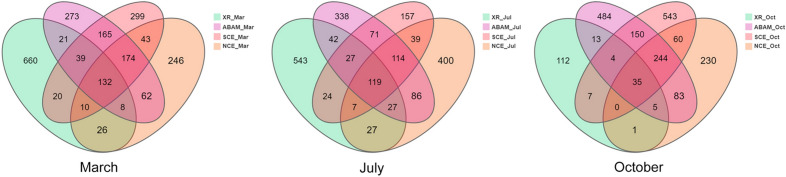
Venn diagrams, representing unique and shared microbial ASVs at 97% identity illustrating the distribution of bacterial taxa among different treatments gathered from data of the three sites in comparison to the natural reef samples over three seasons.

A total of 1179, 1149, 570, and 1059 high-quality 16S rRNA sequences in site A, 1317, 1142, 884 and 320 in site B and 1282, 1090, 1338 and 813 in site C were normalized from the three ABRs models ABAM, SCE, NCE and XR samples respectively (Supplementary Table S2). The Good’s coverage was 99.99–100%, which reveals that the libraries covered almost all the microbial community. With different treatments, the Alpha diversity index of (ABRs) samples was higher than corresponding XR over the three seasons: March, July and October, with the highest obtained with ABAM treatment followed by SCE then NCE which showed the lowest Alpha diversity index. As illustrated in Venn diagrams in Fig. 3; 132, 119 and 35 ASVs were shared among ABAM treatment and the rest of the samples in March, July, and October respectively. All the treatments showed higher number of unique ASVs as well as XR group. Figure 3 also indicates that the unique ASVs belong to ABR samples started to increase over time with corresponding decrease with the XR samples until it reached the maximum in October. It is worth noting that ABAM as well as SCE samples showed the highest unique ASVs. The most important ASVs are the ones that are unique to ABAM. However, the ones that are shared only between ABAM and SCE treatments, is also meaningful as SCE is made of special cement not toxic to the marine environment but lacks the bioactive materials. So, both unique ASVs should be indicators for the specific bacteria restored using the ABR compared to the natural environment. In March, July and October samples, 273 and 163, 338 and 71, 484 and 150 unique ASVs were obtained with ABAM and ABAM/SCE respectively.

The ace value in each sample group ranged from 288 to 535, 144 to 626, 160 to 555 and 37 to 497 with ABAM, SCE, NCE and XR samples respectively (Supplementary Table S2), and the Shannon diversity values for the same samples ranged from 3.42 to 5.19, 2.73 to 5.15, 1.77 to 4.99 and 1.13 to 4.87, respectively.

Within the ABAM groups, the Shannon diversity value in B25 was meaningfully higher than in A10 and C40 in March, but highest in A10 in July and highest in C40 in October (p < 0.01). Within the SCE groups, the Shannon diversity value in B34 was also remarkably higher than in A20 and C55 in March, but highest in C53 in July and highest in A20 in October (p < 0.05). For the NCE, groups, the Shannon diversity value in A59 was clearly lower than B36 and C60 in the three seasons (p < 0.05). For the XR groups, the Shannon diversity value in Bs was slightly lower than As and Cs in March and continued to decrease significantly in July (p < 0.05) while jumped to the highest with slight increase than As and Cs in October (Supplementary Table S2).

**Figure 4:**
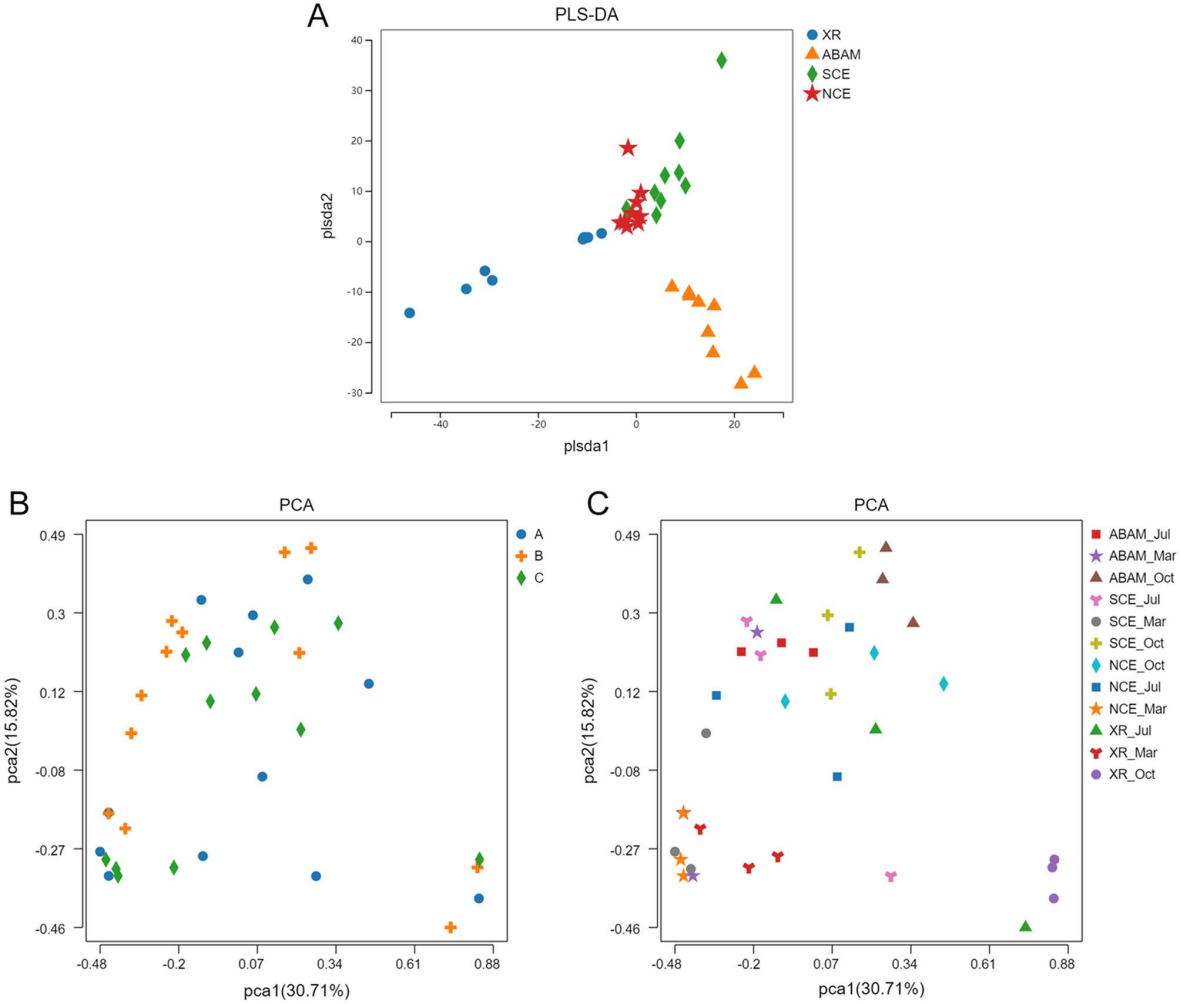
(**A**) Partial least squares Discriminant Analysis (PLS-DA) plots of microbial communities of the three treatments plus the natural reef from the three localities over the three seasons, based on Bray–Curits distances. (**B**,**C**) Principal Component Analysis (PCA) of the different treatments based on ASVs and species abundance.

For Beta diversity, the Partial least squares Discriminant Analysis (PLS-DA) ordination was used to detect the difference in the microbial community patterns between the ABRs with different treatments and natural reef samples (XR) from the three selected localities over the three seasons (Fig. 4A). The Marked differences in taxonomic composition among the XR, from different localities were highlighted. The PLS-DA analysis also reveals a gradual change in natural community composition across the three localities A, B and C (Fig. 4A). It also showed similar microbial community structures in ABAM and SCE samples and tended to group together in contrast to NCE control and XR samples.

A PCA was performed according to the ASVs level to examine the correlation among the microbial community structures on different ABRs and in XR samples collected from the three selected localities over three seasons (Fig. 4B). PCA illustrated that all the samples could be separated based on the phylum abundance data set (Fig. 4C).

### Co-occurrence network analysis

**Figure 5:**
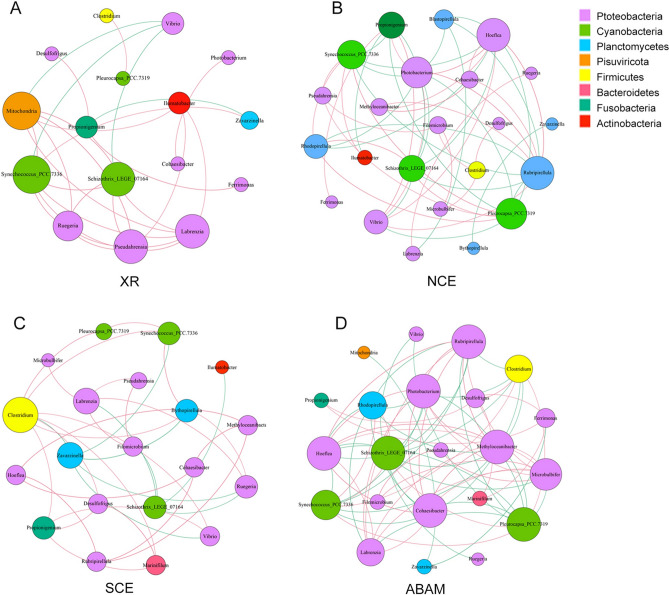
Co-occurrence network analysis at genus level on XR (**A**), NCE (**B**), SCE (**C**), and ABAM (**D**) group. The node colors represent different phyla as shown in the color key. The sizes of genus nodes are proportional to their relative abundance. The red and green edges represent positive and negative correlations, respectively.

The co-occurrence networks of microbial community based on the strong and significant correlations were created to discover synergetic relations among samples from the different treatment models and the XR group (Fig. 5). In total, 71, 34, 51, 27 edges from 22, 20, 23, 16 nodes were identified in the ABAM, SCE, NCE, XR, respectively. The network topological characteristics were calculated to define the complex outlines of connections among microbial genera. The standards of those characteristics, specifically, average degree, average network distance, modularity, and density, are shown in Table 1. The obtained values suggest that microbial communication may be more intensive in ABAM than the rest of the samples.

The positive co-occurrence and negative co-exclusion of microbial genera structure were diverse among ABAM treatment and the others. Negative interactions were higher in ABAM network (47.89%) than in NCE’s (45.10%) and SCE’s (26.47%), signifying higher co-exclusion among genera in ABAM groups. The network nodes of ABAM, SCE, NCE, and XR were similar at the phylum level, whereas Proteobacteria, and Cyanobacteria were the dominant phyla, accounting together for 72.73%, 70%, 65.21%, and 68.75% of the nodes in ABAM, SCE, NCE and XR, respectively.

### Correlation between microbial and macrobenthic community succession on the ABRs

**Figure 6:**
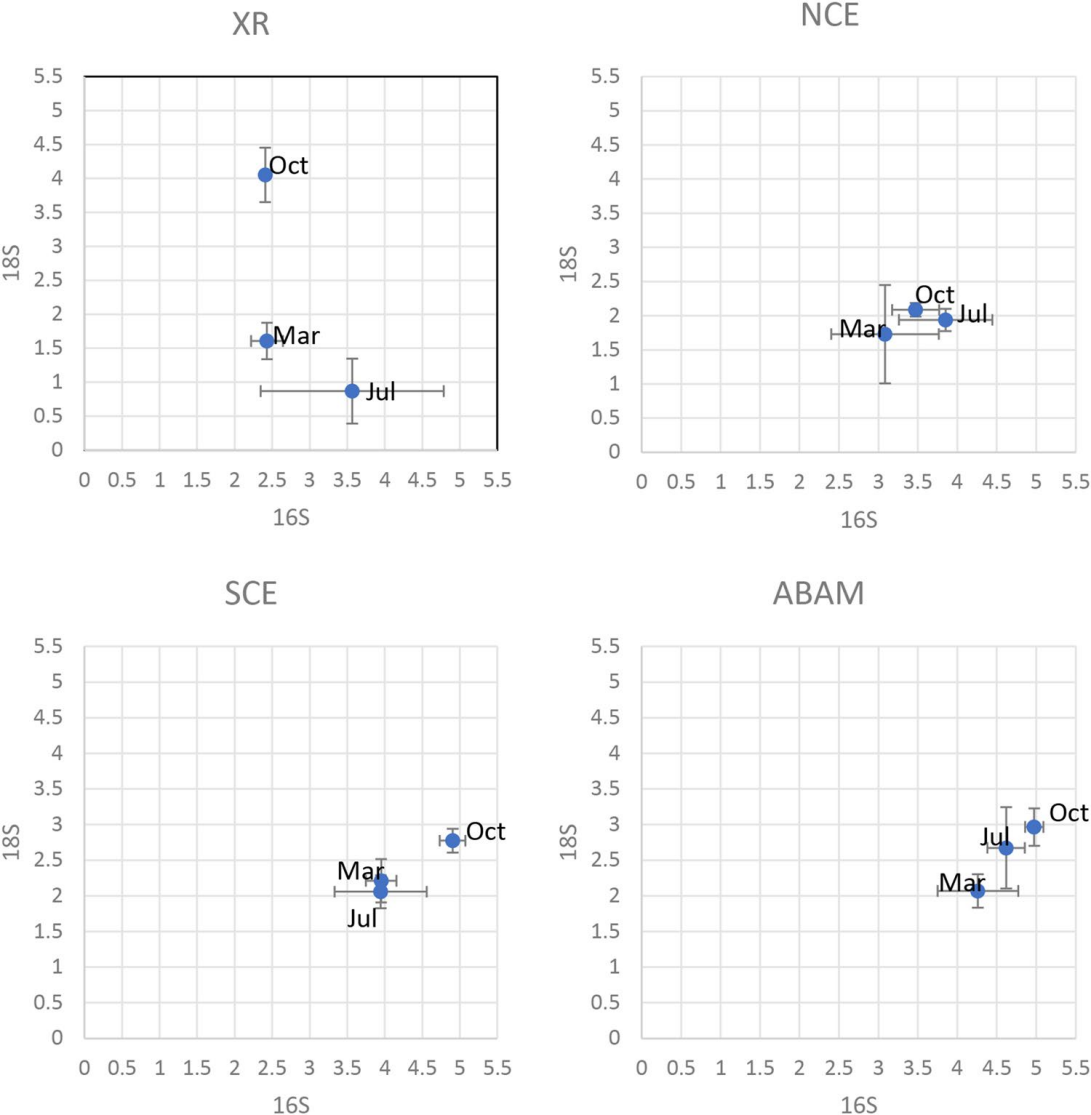
Pearson plots of microbial Shannon index against macrobenthic Shannon index over three seasons.

A total of 18 algal and macrofauna taxa were detected on the ABAM, SCE, NCE models and XR, Rhodophyta (13 taxa), Ochrophyta (3 taxa), and Chlorophyta (2 taxa) (Supplementary Table S3). The macrobenthic patterns were greatly similar on the SCE and ABAM models. However, the species composition that were detected on the ABAM and XR varied over time, especially on October. *Polysiphonia macrocarpa*, *Ulva prolifera* and *Ectocarpus siliculosus* were the most dominant species detected on the ABAM models in March, followed by *Hydrolithon rupestre, Lithophyllum pustulatum* and *Porolithon onkodes* in July while *Gelidium austral* and *Lithophyllum pustulatum* were found as dominant species in October (Supplementary Table S3). Some species were only found on March over the study period, such as *Polysiphonia macrocarpa* (in XR and ABAM) and *Ceratodictyon intricatum* (in XR and SCE). The dominant species also changed in XR: (*Lobophora variegata* and *Ceratodictyon intricatum)*, (*Porolithon onkodes*, *Peyssonnelia* and Symbiochloris sp.* SG-2018*), (*Peyssonnelia novae* hollandiae and *Heterosiphonia pulchra*) were the dominant species in March, July and October, respectively. Linear least-squares regression was performed between the α-diversity of the microbial communities and that of macrobenthos to examine the correlations between them (Fig. 6, Supplementary Table S4). The values of the Shannon diversity index of 16 s showed the highest significant positive correlation with that of 18 s in the ABAM treatment over the three seasons (Pearson’s r = 0.981233), followed by SCE group (r = 0.979893), then NCE with less significant correlation (r = 0.588818) and XR with negative correlation between 18 and 16 s (r = − 0.69256).

## Discussion

In this study, microbial communities covered artificial biological reefs (ABRs)^R^ made up of special concrete structures supported with specific bioactive materials of marine algal origin for sexual coral reef restoration were investigated in comparison to the natural coral ecosystem of three selected sites in Weizhou Island with different coral health status.

**Figure 7:**
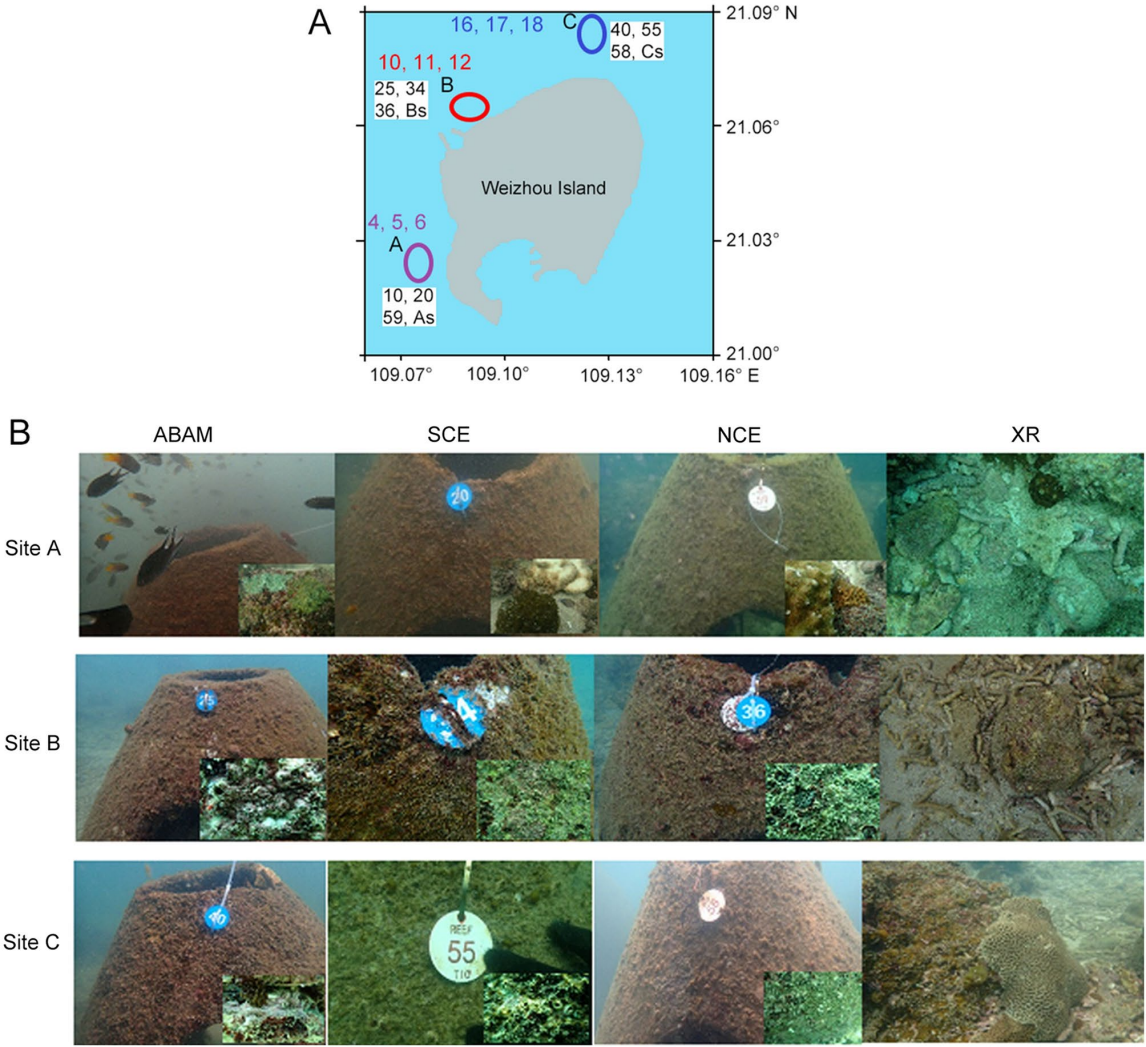
(**A**) A map of Weizhou Island created using Photopea online program (https://www.photopea.com/). The unfilled circles illustrate position of the three chosen sites, A, B and C covering the 9 stations 4, 5, 6, 10, 11, 12, 16, 17, 18. One model of ABRs is placed in each of the indicated stations. Position of deployment and samples from natural reef (XR group) per station are indicated in the white boxes. Purple circle represents site A, station 5 (covering models’ number 10, 20, 59, As), red circle represents site B, station 11 (covering models’ number 25, 34, 36, Bs) and blue circle represents site C, station 17 (covering models’ number 40, 55, 58, Cs). (**B**) A sample of pictures taken during sampling process from both the surface of ABRs and XR from the three sites, credit to Amro Abd-Elgawad.

Our results deliver essential information on microbial structure during the early placement of ABRs and prior to coral larval recruitment as well as two following seasons. Moreover, the macrobenthic community composition has been studied on the installed models in comparison to the natural reef in the three sites as well as in correlation with the developed microbial films over time. The epibenthic communities showed temporal succession on the ABAM models over time. The periphyton including microbes as well as higher algae and invertebrates successively colonized the available suitable substrate surfaces after deployment (Fig. 7). Following, the epibenthic organisms provided the food and shelter for some reef associated habitats including fishes, as can be seen in Fig. 7B. The microbial community abundance on ABAM supported with the bioactive material was significantly different from that on the rest of the controls, with unique biomarkers ranged between 273 and 484 detected within ABAM group over the three seasons. Moreover, on the Phylum level taxonomic distribution, five phyla have been detected: Cyanobacteria, Proteobacteria, Planctomycetota, Bacteroidota, and Actinobacteriota. Microbial community underwent shifts from XR to ABR, with Proteobacteria and Cyanobacteria being the most dominant phyla in all samples set. The dominant phyla on the ABAM treatment as well as the positive control SCE were similar site/season wise. On the other hand, NCE samples showed similar result to the natural environment XR samples while the basic variations were associated with rare phyla representing relative abundance of < 1.0%. The material of substrate used in ARs was previously reported to influence the diversity in former studies^17,18^, in addition to physical and chemical parameters which are known to affect the development of microbial biofilms on the surface of hard substrates, such as; geographic site, substrate positioning, and substrate unevenness^16^. In this study, we report that the Shannon diversity index and the average number of ASVs of the microbial community on the ABAM treatment were the highest followed by SCE compared to the NCE and the natural XR samples. Similar results were obtained with the Shannon index of macrobenthos indicating positive correlation between alpha diversity of microbial community and macrobenthos on the ABAM, and SCE models in contrast to NCE and XR samples which reflects toxicity of the normal cement used to make the control model NCE that leads to similar result to the XR with no restoration. Although there was relative abundance shift among the four types of samples, Cyanobacteria, Proteobacteria and Planctomycetota conserved their dominance on the natural reef which was partially in consistence with earlier studies^6,17,18,21^ who reported Bacteriodota and Proteobacteria as the most dominant phyla. Proteobacteria are developer colonizers^22^, as they represent the most metabolically miscellaneous bacterial phylum which undertakes a wide range of nutritional variability that might be a reason for its widespread. Here, Proteobacteria had slightly increased in abundance in ABAM and SCE models compared to the XR indicating improvement of a useful phylum abundance and it exhibited significant increase over time, indicating better effect of ABAM treatment in attracting functionally required phyla due to the supplement with bioactive materials. Also, Proteobacteria showed faster increase with ABAM and SCE models in site B compared to A and C, indicating that ABAM treatment worked best in site B. This could be due to water eutrophication caused by human impact at different geographical locations of Weizhou Island^23^, which led to the shift in the microbial community pattern and abundance in the XR samples as well as the corresponding ABRs models.

Cyanobacteria: a chief constituent of the biofouling structure, which can yield extracellular polymeric matters to help for better integrative biofilm progress^14,24^, have exhibited unique dominance on all treatments with higher abundance in the ABAM after twelve weeks of deployment, indicating that introduction of any new substrate can attract better Cyanobacteria compared to the natural reef which has damaged substrate indicating human impact rather than environmental stress, with ABAM showing better attraction of Cyanobacteria because of added supplementary materials. Later, ABAM and SCE models showed faster decrease of Cyanobacteria in site B than in site C. We speculate that the Cyanobacteria either occupied the ABRs just after their installment or undertook Cyanobacterial blooming over different seasons and it showed decline in abundance over time when its role in integrating the first biofilm has ended and started to decline for better colonization with the next beneficial phyla at that specific time such as Proteobacteria as shown in the result. This hypothesis also goes well with the fact that ABAM treatment showed the best control on Cyanobacteria to act in this manner. This might also explain the fact that site B which is known for having the most deteriorating coral reef status had the least Cyanobacteria abundance in March compared to the rest of the sites. This could be interpreted that the stressful surrounding has led to less cyanobacteria to colonize the available substrates and hence, the consequence beneficial phyla. However, it’s difficult to confirm the effect of Cyanobacteria on the successful succession of microbial taxa because of lack of species identification. Moreover, the ABAM treatment had greater influence on attracting functionally important bacterial taxa for consequent marine habitat restoration.

On the Genus-level, in March, XR samples exhibited highest diversity with As followed by Cs then Bs samples. Accordingly, ABAM retained best diversity with A10, followed by C40 and B25. The NCE control showed more consistency in the three sites and less diversity compared to ABAM and positive control SCE, but still higher than that of the XR which could be explained by the toxicity of the normal cement used in the NCE compared to the ABAM and SCE models, as well as the poor quality of the natural reef in the damaged reef. In July, *Vibrio* started to appear significantly in all samples with the highest abundance in the XR and NCE samples. Consequently, diversity and abundance of the rest of the phyla have been greatly affected^28^. In contrast, later *Vibrio* was effectively controlled by ABAM treatment and SCE with consistent increase in diversity and abundance of the rest of the phyla. One of the important Genus is *Rugeria* which exhibited consistent abundance with ABAM and SCE compared to NCE and XR samples. This species is known to offer probiotic benefits to corals, such as B12 vitamin production and protection against some coral pathogens like *Vibrio coralliilyticus*^25^. This clearly indicates that ABAM models have attracted bacterial genera known for their defense against coral pathogens, and thus explains better tolerance of microbial biofilms on this model compared to the control models and the natural reef. In October, *Vibrio* continued to share the dominance with *Propioneginium* in the XR group while diminished in the rest of the samples. In most cases, ABAM treatment showed close results to SCE control treatment and that NCE treatment was closer to the XR result indicating high toxicity of the type of cement in the NCE treatment and explains the declined health in site B and A. Thus, ABAM has stable symbioses with beneficial bacteria. In contrast, the bacterial community exhibited flexibility in the XR samples with old biofilm and harmful microbial community. For example, potentially pathogenic *Vibrio* (ASV827, ASV539, ASV170, and ASV375) showed high relative abundance in XR and NCE groups, of which existence may cause rise in gene expression linked to virulence^26,27^. Some research reported that the relative abundance of *Vibrio* as heterotrophic bacteria increases in coral reefs in response to high temperature, water contamination^26,27^, or due to coral diseases^28–31^. Our study does not only highlight the importance of abundance but also considering the persistence to determine functionally essential bacteria over time linked with ABR and coral reefs. Here, two bacterial phylotypes were highly ubiquitous and abundant among all community structures (Proteobacteria was highly persistent across site/season levels while Cyanobacteria was persistent across site only). PCA illustrated that all the samples could be separated based on the phylum abundance data set (Fig. 4C), meaning that these three treatments had clearly different bacterial phyla abundance and they were different from the XR indicating that available substrates have direct effect on structuring the microbial pattern. In general, phylum analysis indicated that Proteobacteria replaced Cyanobacteria as the dominant clade as time going. Location wise, site B exhibited the highest level of abundance of Proteobacteria in March and July, highest Plactomycetota and Firmicutes in October. On the other hand, the Genus-level, of XR samples taken in March exhibited variations abundance among different sites, where site B and C showed very low diversity indicating less significance of diversity compared to abundance in the early stages prior to coral recruitment.

In general, Macrobenthic community development showed a positive correlation with the microbial diversity structures on the ABAM surfaces (Fig. 7B), indicating successful restoration of a whole coral reef ecosystem using the ABR.

As presented in Fig. 5, the node diameter of Protebacteria in the ABAM group is the largest, indicating that Protebacteria is higher in abundance and more closely related to other genera in the ABAM group than other groups. In the network diagram of ABAM, Mitochondria appears and Ilumatobacter disappears, and they are the only genera that appear and disappear respectively in Pisuvericota and Actinobacteria. That is to say, after ABAM treatment, Pisuvericota gradually played an important role in the community, while the relationship of Actinobacteria and other genera gradually weakened. It is the Mitochondria and Ilumatobacter that are most sensitive to ABAM. Compared with XR and NCE group, Marinifilum and Clostridium are more related to other genera in ABAM and SEC group. In the four groups, there are three genera of cyanobacteria with greater correlation, but the difference is that in the ABAM group, the negative correlation between cyanobacteria and other genera is greater. Our experiment in situ is applying the natural exposure of our models to the dynamic environmental variables that might have affected the microbial films such as ocean current and physico-chemical properties of the surrounding seawater. Hence, reflecting the natural co-occurrence of the micro and microbenthic communities in the enhanced environment.

## Conclusions

Coral reef microbiome has multi essential functions in managing life of marine habitat; including larval settlement and metamorphosis, biofilm colonization, and pathogen domination under extreme environmental conditions, thus, controlling developed acclimatization of the whole coral reef ecosystems. We here introduce artificial structure, ABAM models supported with bioactive materials which have shown enhanced microbial biofilm preior to coral larval recruitment as well as following two seasons, including control over *Vibrio* which was extensively abundant on the XR samples as well as the control models. This finding was associated with the abundance of *Rugeria* genus on ABAM models which draws attention towards involvement of *Rugeria* in inhibiting the *Vibrio* on the ABAM models, which is a question to answer in the future research. On ABAM, macrobenthic genera were more closely related to each other and are more negatively correlated. As most of the interactions between micro and macrobenthic communities up to date are done in the laboratories, and taking into account the complex relationship between both of them, a combined experiment includes in situ with more interrupting impacts and ex situ experiments is needed to better elucidate the co-occurrence analysis in the future. Thus, we here report a successful trial to sustainable development of healthy microbial and macrobenthic community on Artificial Biological Reef structures (ABRs)^R^ supported with bioactive material for marine habitat larval attraction, associated with severely, moderately, and slightly damaged coral reef in three coral sites in Weizhou Island with different health status.

## Materials and methods

### Study site, ABRs construction, deployment and sample collection

The study area is located 21 km south of Beihai city, China, that outspreads in Beibu Gulf. Three sites of coral reef, A, B, and C (between 21° 1.3966′ N, 109° 4.6977′ E and 21° 5.008′ N, 109° 7.488′ E) were chosen using Global Positioning System (GPS) (Fig. 7A) to install nine ABRs structures (three in each site) for coral reef sexual restoration at end of December 2020. The construction models were placed about 2 m apart in all sites. Chosen sites are described according to coordinates, coral reef structure, coral coverage and noncoral coverage. Information for sampling locations, is shown in Table 3.

**Table 3:** Site description: Three sites A, B, and C are described according to coordinates, coral reef structure, coral coverage and noncoral coverage.

Site no.	Station no.	N	E	Coral reef structure	Coral coverage %	Noncoral coverage %
A	4	21° 1.3966′ N	109° 4.6977′ E	Sandy reef with *Pavona* patches	53	47
	5	21° 1.635′ N	109° 4.807′ E			
	6	21° 1.246′ N	109° 4.632′ E			
B	10	21° 3.9070′ N	109° 5.5701′ E	Sandy reef	7	93
	11	21° 3.978′ N	109° 5.748′ E			
	12	21° 3.910′ N	109° 5.440′ E			
C	16	21° 5.0074′ N	109° 7.5813′ E	Sandy reef with *Pavona* patches	80	20
	17	21° 4.916′ N	109° 7.668′ E			
	18	21° 5.008′ N	109° 7.488′ E			

**Figure 8:**
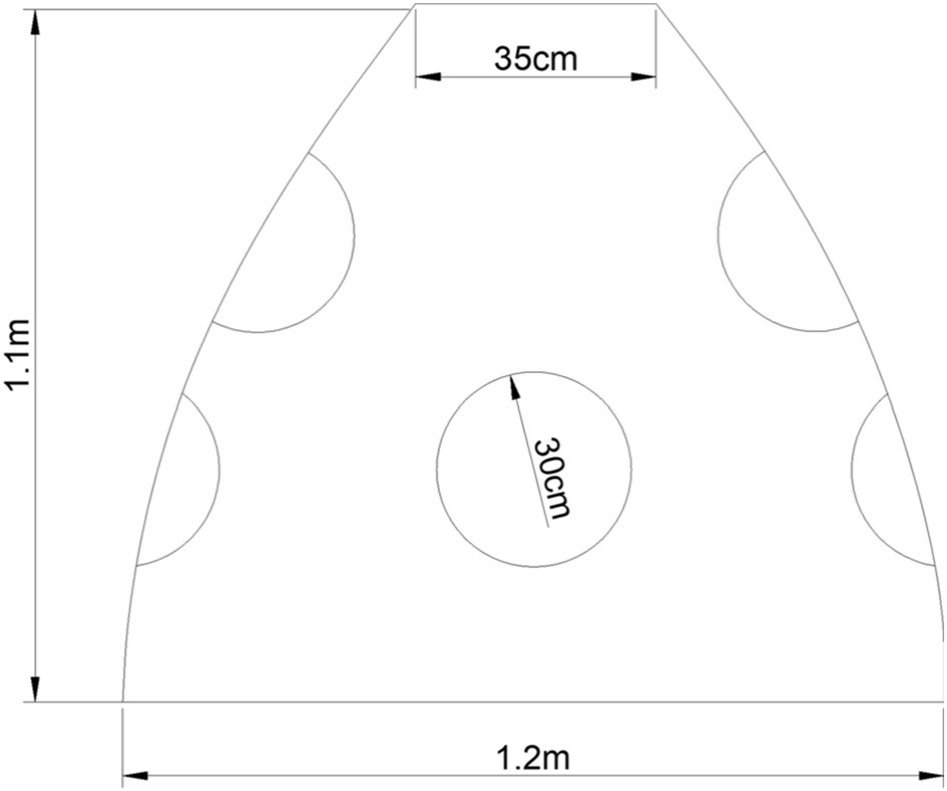
Outline diagram of ABR structure.

Semiartificial substrate composed of a special cement supported with environment-friendly material made of variety of seaweed extracts marine algal source (patency registration No. 202111290154.5, unpublished data) was used to construct a conical like artificial biological reef (ABR) as shown in Fig. 8, to mediate the attraction and survival of coral and other marine habitat larval recruitment and metamorphosis designated as (ABAM, supplemented with bioactive material), (SCE, made up of seawater cement and used as a positive control), (NCE, made up of normal cement and used as a negative control). These designs are constructed to work for sexual restoration of damaged coral reef ecosystem.

A total of thirty-six samples were collected over three seasons in 2021; Spring (end of March) just prior to coral larval spooning in April, Summer (July), and Autumn (October), twelve from each season; three from the sediment of natural reef of the three sites (XR group) and nine from the surface of the ABRs installed in the three sites; three from ABAM, three from SCE and three from NCE model structures (Table 2, Fig. 7). Samples from the natural reef As, Bs, and Cs were collected in triplicates. Three replicate’s line transects were laid parallel to the shoreline at two depths (3.5 and 7.5 m) representing the shallow and deep zones of the reef front. Samples from the surface of the ABRs were collected in 8 replicates to ensure covering of the whole surface of the ABR. The eight replicates for each sample are mixed in one tube for later comparison and assessment of microbial community composition (XR groups) VS the (ARs group) using 16S High throughput sequencing analysis and also for 18S High throughput to detect succession and diversity of macrobenthos on the ABRs and XR. Samples were collected using single line transect^32^ with scuba diving utilities using a hammer, chisel, and gloves. Samples were kept in ice then stored at – 20 °C. The average of the replicates of each line transects represents the percentage values for each site.

### Water quality measurements of the local environment of Weizhou Island

The physical and chemical parameters indicating water quality in Weizhou Island have been collected from previous resources. The average annual SST in Weizhou Island is 24.62 °C. The mean annual air temperature is 22.6 °C; the mean annual precipitation is 1380.2 mm; mean wind speed is 4.8 m/s^33^. Monthly average salinity at Weizhou Island ranges from 31.17 to 32.66 over 20 years^34^. The dissolved oxygen saturation is above 90 and no anoxia was spotted through the year^35^. Weizhou Island is characterized of rainfall, and ambient light. The acidification of the water ranges between 8.0 and 8.23. Ocean is clear, with transparency ranging from 3.0 to 10.0. Moreover, there is cyclonic movement all across the year^33^.

### DNA extraction, PCR amplification and high-throughput sequencing

DNA extraction was conducted using CTAB protocol and Zymo DNA Clean & Concentrator kit (Zymo Research Corp, Irvine, USA) following the manufacturer’s protocol. The samples were suspended in 600 μL of DNA lysis buffer and 10 μL proteinase K (Takara, Japan) at 55 °C for 48 h. To the suspension, 82.5 μL of 10% w/v CTAB (acetyl/hexadecyl trimethyl ammonium bromide, in 0.7 M NaCl) and 82.5 μL of 5 M NaCl, were added and incubated at 55 °C for 10 min. Next, Chloroform (600 μL) was added then vortexed and centrifuged for 10 min at 11,000*g*. The upper layer was removed to a clean tube and the purification step was carried out using DNA Clean and Concentration Kit (ZYMO Research ZRC000570, USA) following the manufacturer’s protocol: The primers 515F (5′-GTGCCAGCMGCCGCGG-3′)/907R (5′-CCGTCAATTCMTTTRAGT-3′)^36^ targeting the V4–V5 domain of prokaryotic SSU rDNA were used. The primers TAReuk454FWD1 (50-CCAGCASCYGCGGTAATTCC-30) and TAReukREV3 (50-ACTTTCGTTCTTGATYRA-30)^37^ targeting the V4–V5 domain of eukaryotic SSU rDNA were also used. Polymerase chain reaction was performed following the protocol of Ammon et al.^38^ with minor changes. The Ex Taq PCR-kit (Takara Bio, Japan) was used according to protocol with primer concentration of 0.2 μM. The PCR reaction involved, denaturation step for 34 cycles at 95 °C for 30 s, 56 °C for 30 s, annealing at 72 °C for 1 min 30 s and extension at 72 °C for 5 min. Amplicons were purified then libraries were performed using the Ion Plus Fragment Library Kit 48 rxns (Thermo Scientific, Walthman, MA, USA). The 16 s rDNA and 18 s rDNA libraries were sequenced on an Illumina MiSeq platform (Illumina, San Diego, USA) using a paired-end (2 × 250 bp) HiSeq Reagent Kit following manufacturer’s instructions in Beijing Genomics Institute (BGI, Beijing, China). Generated sequences were assessed and analyzed using DADA2 (Divisive Amplicon Denoising Algorithm^39,40^ software package in R. The sequences were filtered and cleaned using the DADA2 workflow^39,40^ amplicon sequence variants (ASVs) determination. The ASVs were aligned to the SILVA rRNA database^41^; https://www.arb-silva.de/) for 16 s sequencing, and PR2 rRNA database^42^ (http://ssu-rrna.org/)) for 18 s sequencing. Illumina next-generation DNA sequences were deposited in the Sequencing Read Archive (SRA) of the National Centre for Biotechnology Information (NCBI) under BioProject accession PRJNA851196 ID: 851196, Biosample accessions SAMN29390395–SAMN29390466.

### Statistics and bioinformatic analysis

The α-diversity of bacterial community composition including the ASV richness, ACE^43^, Chao1^44^, Pielou index^45^, Simpson diversity^46^, Shannon diversity^47^, rarefaction curves were analyzed using R^48^ using the package vegan^49^. Venny’s on-line website (https://bioinfogp.cnb.csic.es/tools/venny/index.html) was used for conducting the venn diagrams. The β-diversity analyses of bacterial Bray–Curtis dissimilarity was performed using the vegdist function in vegan package. To detect taxonomic variability among different treatments and XR samples, a least discriminant analysis (LDA) was conducted to recognize biomarkers (threshold > 4.0)^50^. The co-occurrence networks were performed by calculating the Spearman’s rank correlations with the psych package based on the relative abundance of genera, with |ρ| greater than 0.6 and a p value of less than 0.05, using Gephi (v. 0.9.2)^51^. The linear least-squares regression mode was used to identify the correlation between α-diversity of both macrobenthos and microbial community (ASV richness and Shannon index) and to assess the persistence of the relative abundance of dominant phyla over time.

### Patents

ABRR was registered for Chinese patency with application no. 202111290154.5.

## Supplementary Information

Supplementary Tables.

## Data Availability

Sequence data is deposited to NCBI data base under BioProject accession PRJNA851196, Biosample accessions SAMN29390395–SAMN29390466 and will be available for reviewers before publication.
