# Author Correction: Microbial community shift on artificial biological reef structures (ABRs) deployed in the South China Sea

**DOI:** 10.1038/s41598-023-32201-7

**Published:** 2023-04-25

**Authors:** Hala F. Mohamed, Amro Abd‑Elgawad, Rongshuo Cai, Zhaohe Luo, Lulu Pie, Changan Xu

**Affiliations:** 1 Third Institute of Oceanography, Ministry of Natural Resources, Xiamen, 361005 People’s Republic of China; 2 Botany & Microbiology Department, (Girls Branch), Faculty of Science, Al-Azhar University, Cairo, Egypt; 3 Tourism Developing Authority, Central Administration for Environmental Affairs, Cairo, Egypt

Correction to: *Scientific Reports* 10.1038/s41598-023-29359-5, published online 01 March 2023

The original version of this Article contained an error in the order of the Figures. Figure 1 was published as Figure 3, Figure 3 was published as Figure 1, Figure 4 was published as Figure 8, Figure 5 was published as Figure 7, Figure 7 was published as Figure 5, and Figure 8 was published as Figure 4. The Figure legends were correct.

The original Figures 1, 3, 4, 5, 7, 8 and accompanying legends appear below.

In addition, the Funding section contained an error.

“This research was funded by the National Key Research and Development Program of China [2017YFA0604902], The Scientific Research Foundation of the Third Institute of Oceanography, MNR [2020002, 2020001], China APEC Foundation [HV01-190101(1)], Xiamen, and Marine and Fishery Development Special Foundation [19CZP011HJ08].”

now reads:

This research was funded by the National Key Research and Development Program of China [2017YFA0604902], The Scientific Research Foundation of the Third Institute of Oceanography, MNR [2020002, 2020001], the High-level Foreign Experts Funding Program of China (G2021055005L), China APEC Foundation [HV01-190101(1)], Xiamen, and Marine and Fishery Development Special Foundation [19CZP011HJ08].

**Figure 1:**
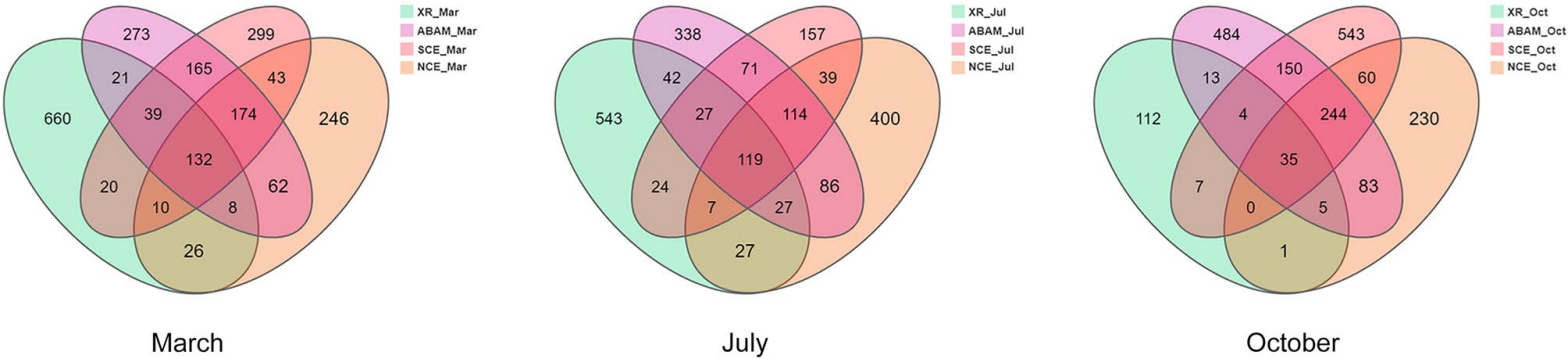
Temporal dynamics of the relative abundances of top bacterial phyla and genera of the three sampling treatments (ABAM, SCE and NCE) Plus the samples from the natural environment (XR) for the three different localities. (**A**–**C**) Relative abundance of key bacterial Phyla in March, July, and October respectively. (**D**) Relative abundance of key bacterial Phyla of total bacteria grouped for all three localities with the different treatments plus the XR. (**E**–**G**) Relative abundance of dominant bacterial genera in March, July, and October respectively. (**H**) Relative abundance of key bacterial Genera of total bacteria grouped for all three localities with the different treatments plus the XR (**I**) Heat map of the relative abundance of the 20 most abundant classes over time.

**Figure 3:**
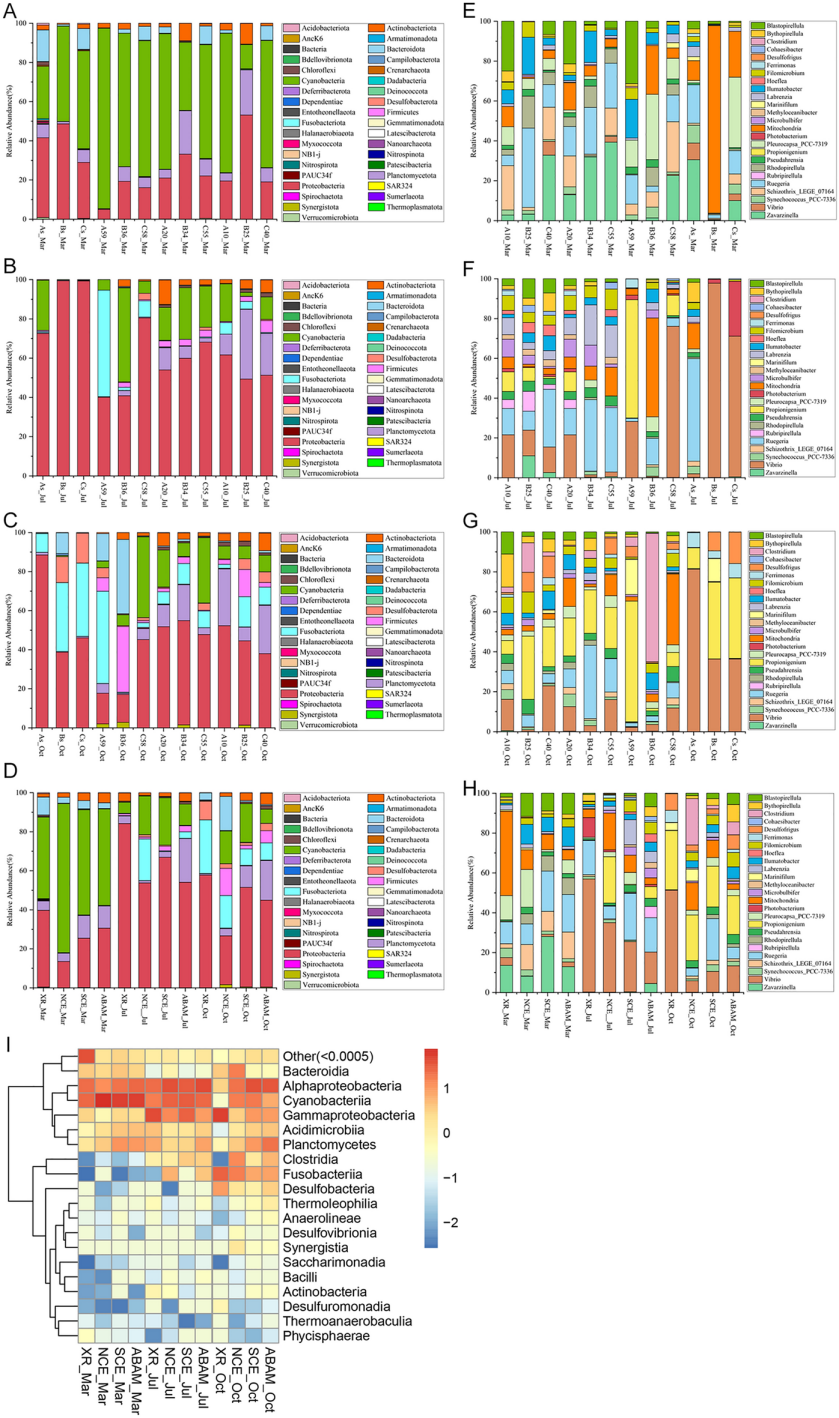
Venn diagrams, representing unique and shared microbial ASVs at 97% identity illustrating the distribution of bacterial taxa among different treatments gathered from data of the three sites in comparison to the natural reef samples over three seasons.

**Figure 4:**
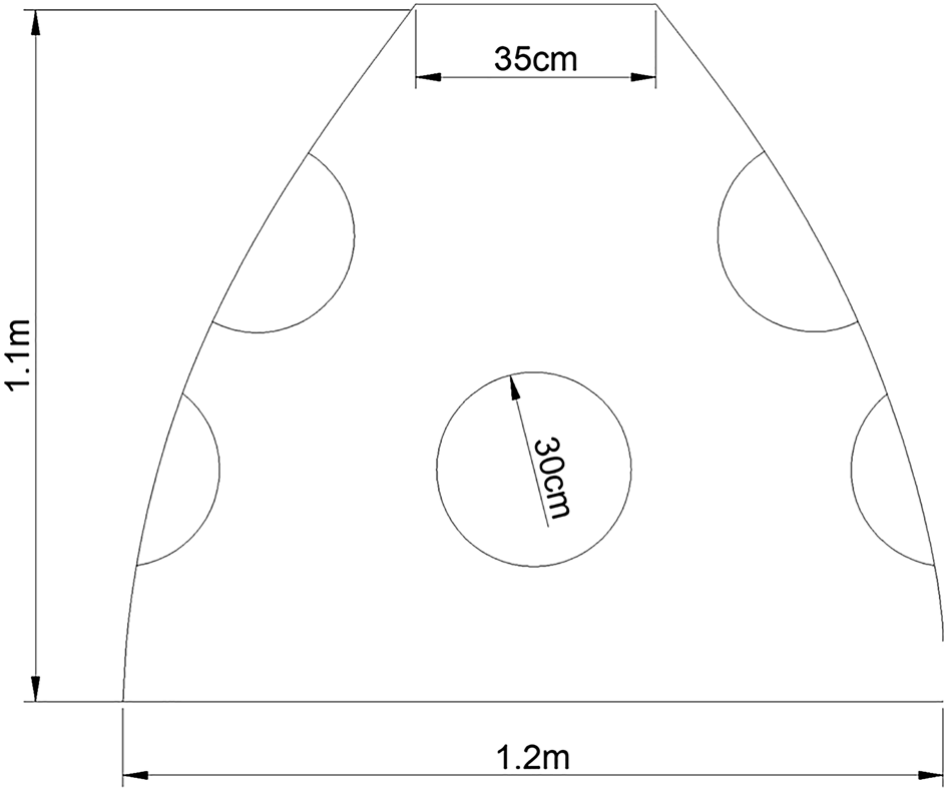
(**A**) Partial least squares Discriminant Analysis (PLS-DA) plots of microbial communities of the three treatments plus the natural reef from the three localities over the three seasons, based on Bray–Curits distances. (**B**,**C**) Principal Component Analysis (PCA) of the different treatments based on ASVs and species abundance.

**Figure 5:**
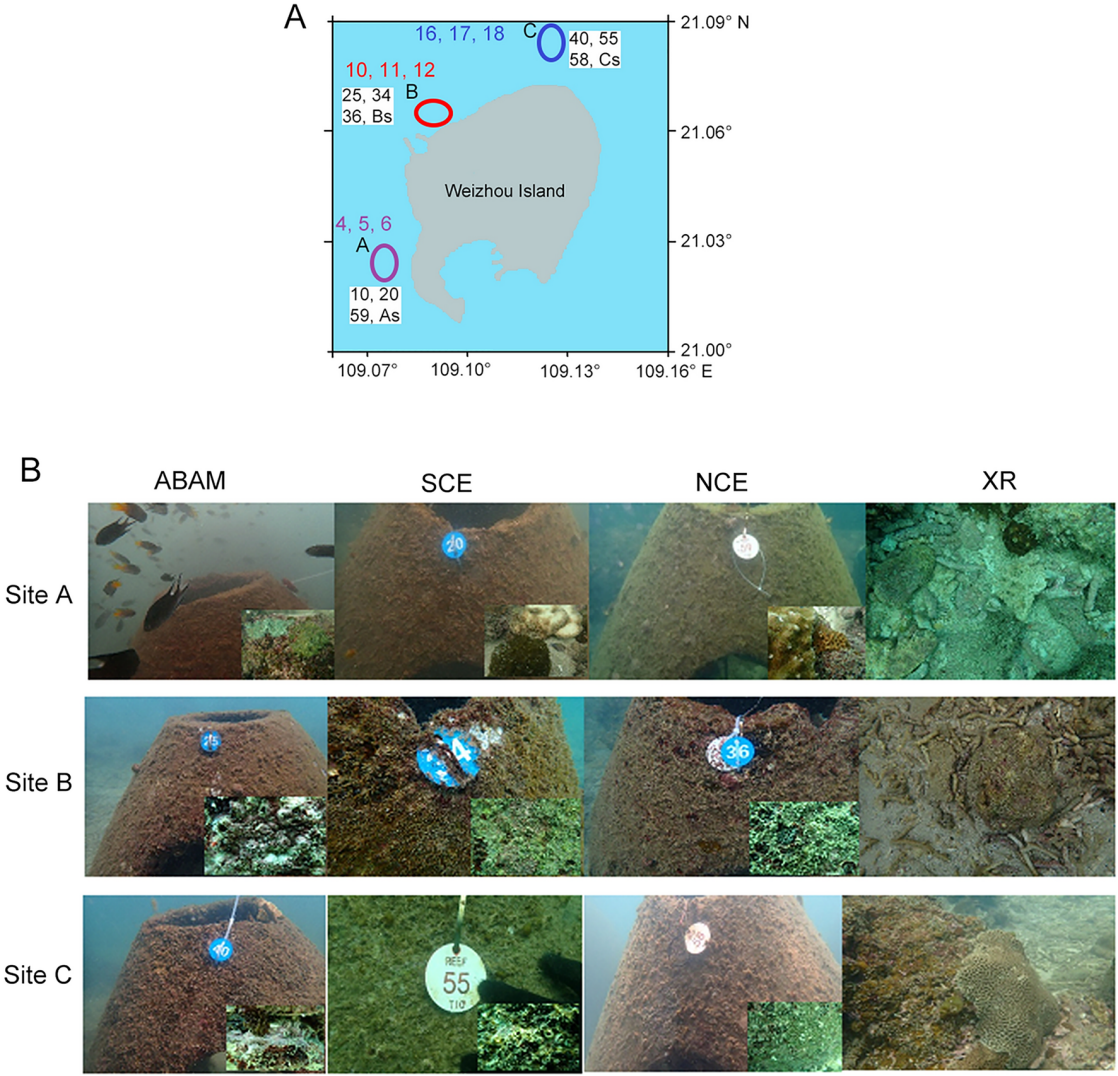
Co-occurrence network analysis at genus level on XR (**A**), NCE (**B**), SCE (**C**), and ABAM (**D**) group. The node colors represent different phyla as shown in the color key. The sizes of genus nodes are proportional to their relative abundance. The red and green edges represent positive and negative correlations, respectively.

**Figure 7:**
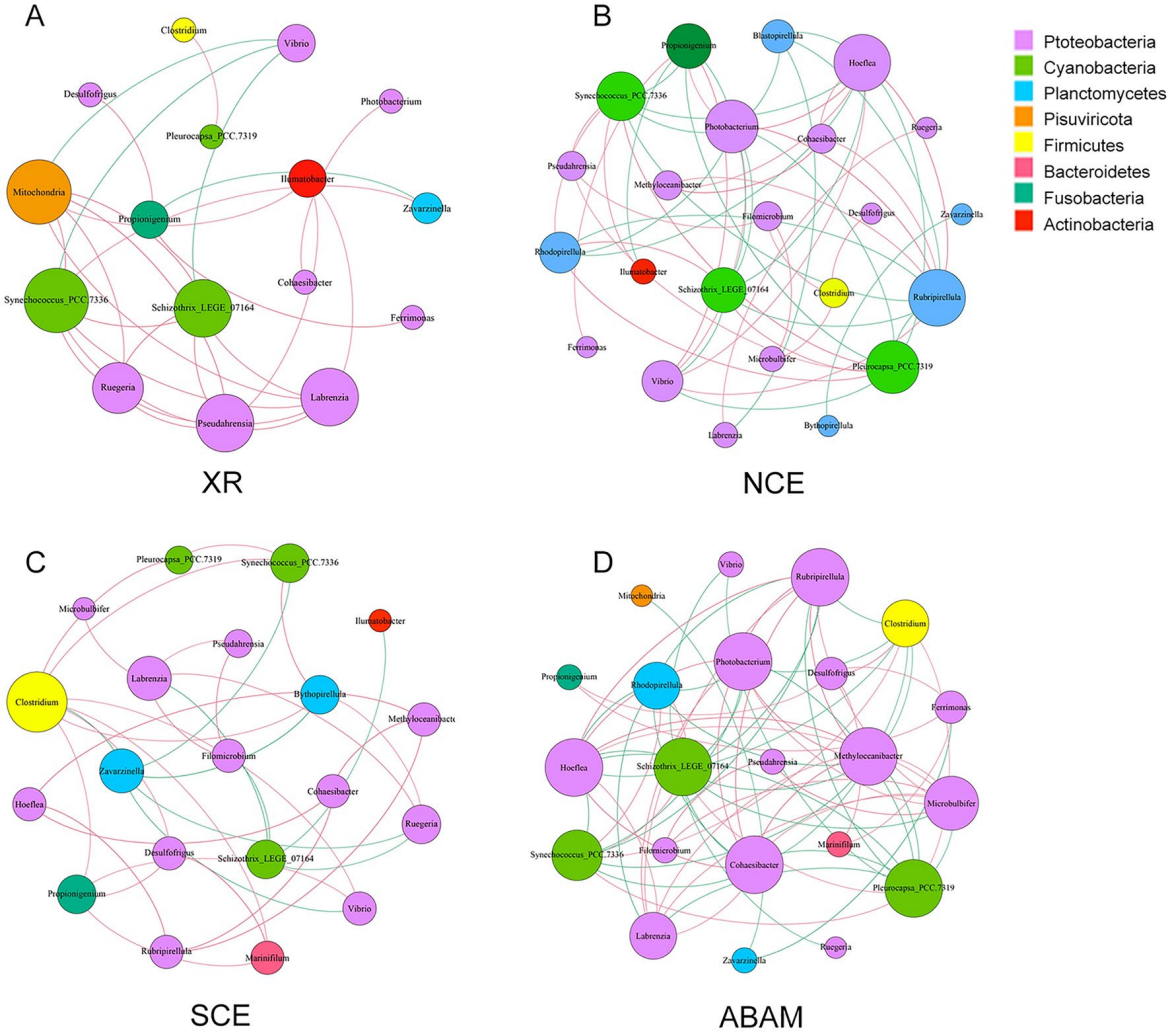
(**A**) A map of Weizhou Island created using Photopea online program (https://www.photopea.com/). The unfilled circles illustrate position of the three chosen sites, A, B and C covering the 9 stations 4, 5, 6, 10, 11, 12, 16, 17, 18. One model of ABRs is placed in each of the indicated stations. Position of deployment and samples from natural reef (XR group) per station are indicated in the white boxes. Purple circle represents site A, station 5 (covering models’ number 10, 20, 59, As), red circle represents site B, station 11 (covering models’ number 25, 34, 36, Bs) and blue circle represents site C, station 17 (covering models’ number 40, 55, 58, Cs). (**B**) A sample of pictures taken during sampling process from both the surface of ABRs and XR from the three sites, credit to Amro Abd-Elgawad.

**Figure 8:**
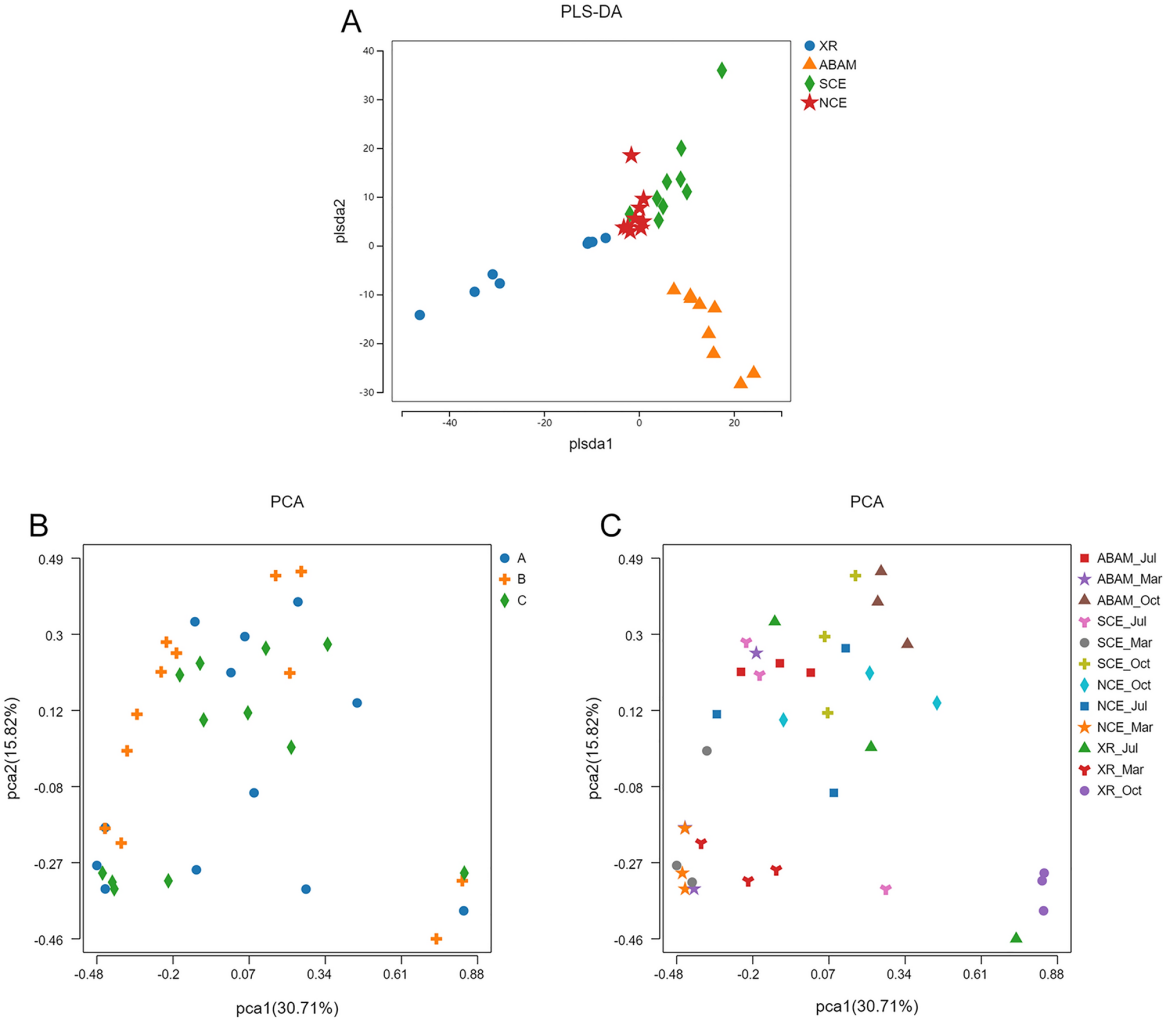
Outline diagram of ABR structure.

The original Article has been corrected.

