# Supplementary material for: Microbial community shift on artificial biological reef structures (ABRs) deployed in the South China Sea
Source: Sci Rep. 2023 Mar 1;13:3456. doi: 10.1038/s41598-023-29359-5 (PMC9977770; doi:10.1038/s41598-023-29359-5)
Supplement: Supplementary file 1 — Supplementary Tables. [file 41598_2023_29359_MOESM1_ESM.docx]

**Table S1: Statistics of data filtering.**

| Sample  name | Total Pairs Read_num | Filtered Read_num | Denoised Read_num | Merged Read_  num | Non-Chimeric Read_num | Tag_num | asv_num |
| --- | --- | --- | --- | --- | --- | --- | --- |
| A10_Mar | 67744 | 64656 | 95.44 | 60604 | 37537 | 28870 | 356 |
| A20_Mar | 67991 | 65060 | 95.69 | 60505 | 40283 | 28805 | 380 |
| A59_Mar | 66978 | 64169 | 95.81 | 62376 | 48272 | 34412 | 160 |
| As_Mar | 67067 | 64425 | 96.06 | 58188 | 44444 | 40491 | 497 |
| B25_Mar | 67311 | 64435 | 95.73 | 58276 | 30410 | 22848 | 419 |
| B34_Mar | 67155 | 63987 | 95.28 | 59279 | 37815 | 29176 | 419 |
| B36_Mar | 67231 | 64317 | 95.67 | 61217 | 42318 | 33496 | 337 |
| Bs_Mar | 67314 | 64521 | 95.85 | 62011 | 50861 | 39986 | 194 |
| C40_Mar | 67632 | 63981 | 94.6 | 60057 | 41769 | 29699 | 418 |
| C55_Mar | 67405 | 64487 | 95.67 | 59443 | 39261 | 26267 | 438 |
| C58_Mar | 67521 | 64487 | 95.51 | 60285 | 38913 | 27124 | 415 |
| Cs_Mar | 67785 | 63968 | 94.37 | 58420 | 41943 | 30765 | 356 |
| A10_Jul | 68177 | 63066 | 92.5 | 59820 | 32985 | 28360 | 288 |
| A20_Jul | 68739 | 62371 | 90.74 | 60529 | 40122 | 33662 | 144 |
| A59_Jul | 68391 | 63828 | 93.33 | 61258 | 38750 | 31515 | 202 |
| As_Jul | 67944 | 63409 | 93.33 | 59918 | 35700 | 30769 | 489 |
| B25_Jul | 67650 | 62839 | 92.89 | 60804 | 49685 | 48238 | 486 |
| B34_Jul | 68950 | 63317 | 91.83 | 60284 | 31033 | 23804 | 272 |
| B36_Jul | 68688 | 63863 | 92.98 | 61175 | 36481 | 29582 | 257 |
| Bs_Jul | 68474 | 63138 | 92.21 | 62288 | 44412 | 36272 | 37 |
| C40_Jul | 67958 | 63324 | 93.18 | 59998 | 30579 | 25536 | 376 |
| C55_Jul | 68246 | 63342 | 92.81 | 60231 | 33865 | 27784 | 329 |
| C58_Jul | 68476 | 64109 | 93.62 | 63063 | 45785 | 38472 | 555 |
| Cs_Jul | 68652 | 64433 | 93.85 | 61089 | 34590 | 28316 | 372 |
| A10_Oct | 68028 | 63247 | 92.97 | 60409 | 37378 | 34138 | 535 |
| A20_Oct | 67454 | 62882 | 93.22 | 61970 | 50354 | 49123 | 625 |
| A59_Oct | 67935 | 62043 | 91.33 | 59912 | 41100 | 37110 | 208 |
| As_Oct | 68681 | 62326 | 90.75 | 60961 | 38720 | 25227 | 73 |
| B25_Oct | 68005 | 63633 | 93.57 | 60104 | 33499 | 30351 | 412 |
| B34_Oct | 67216 | 62734 | 93.33 | 58923 | 32323 | 28202 | 451 |
| B36_Oct | 67601 | 63044 | 93.26 | 61126 | 43258 | 38044 | 290 |
| Bs_Oct | 67544 | 62994 | 93.26 | 61529 | 41886 | 31466 | 89 |
| C40_Oct | 68038 | 63824 | 93.81 | 60668 | 37826 | 35017 | 488 |
| C55_Oct | 68119 | 63884 | 93.78 | 60807 | 35908 | 31141 | 323 |
| C55_Oct | 67226 | 62909 | 93.58 | 59996 | 39491 | 37039 | 368 |
| Cs_Oct | 67865 | 61607 | 90.78 | 60243 | 42166 | 33803 | 85 |

**Table S2: The average of Alpha diversity indices of bacterial communities in the ABRs treatments plus XR.**

| Sample_name | sobs | chao | ace | Shannon | Simpson | Coverage |
| --- | --- | --- | --- | --- | --- | --- |
| A10_Mar | 356 | 356 | 356.00 | 4.17 | 0.07 | 1 |
| A20_Mar | 380 | 380 | 380.00 | 3.76 | 0.14 | 1 |
| A59_Mar | 160 | 160 | 160.24 | 1.77 | 0.52 | 0.999971 |
| As_Mar | 497 | 497 | 497.19 | 2.43 | 0.43 | 0.999975 |
| B25_Mar | 419 | 419 | 419.00 | 5.19 | 0.01 | 1 |
| B34_Mar | 419 | 419 | 419.00 | 4.36 | 0.08 | 1 |
| B36_Mar | 337 | 337 | 337.00 | 3.43 | 0.17 | 1 |
| Bs_Mar | 194 | 194 | 194.00 | 2.07 | 0.26 | 1 |
| C40_Mar | 418 | 418 | 418.00 | 3.42 | 0.23 | 1 |
| C53_Mar | 438 | 438 | 438.00 | 3.74 | 0.15 | 1 |
| C60_Mar | 415 | 415 | 415.00 | 4.05 | 0.07 | 1 |
| Cs_Mar | 356 | 356 | 356.00 | 2.80 | 0.30 | 1 |
| A10_Jul | 288 | 288 | 288.00 | 4.46 | 0.03 | 1 |
| A20_Jul | 144 | 144 | 144.00 | 2.73 | 0.17 | 1 |
| A59_Jul | 202 | 202 | 202.00 | 3.00 | 0.14 | 1 |
| As_Jul | 489 | 489 | 489.20 | 4.87 | 0.03 | 0.999967 |
| B25_Jul | 486 | 486 | 486.00 | 4.31 | 0.07 | 1 |
| B34_Jul | 272 | 272 | 272.19 | 4.46 | 0.03 | 0.999958 |
| B36_Jul | 257 | 257 | 257.00 | 3.57 | 0.13 | 1 |
| Bs_Jul | 37 | 37 | 37.00 | 1.13 | 0.48 | 1 |
| C40_Jul | 376 | 376 | 376.17 | 5.09 | 0.01 | 0.999961 |
| C53_Jul | 329 | 329 | 329.21 | 4.65 | 0.02 | 0.999964 |
| C60_Jul | 555 | 555 | 555.00 | 4.99 | 0.02 | 1 |
| Cs_Jul | 372 | 372 | 372.00 | 4.70 | 0.03 | 1 |
| A10_Oct | 535 | 535.111111 | 535.34 | 5.18 | 0.02 | 0.999941 |
| A20_Oct | 625 | 625.75 | 625.46 | 5.15 | 0.02 | 0.999939 |
| A59_Oct | 208 | 208 | 208.00 | 2.99 | 0.20 | 1 |
| As_Oct | 73 | 73 | 73.23 | 2.32 | 0.16 | 0.99996 |
| B25_Oct | 412 | 412.3 | 412.65 | 4.79 | 0.03 | 0.999901 |
| B34_Oct | 451 | 451 | 451.00 | 4.99 | 0.02 | 1 |
| B36_Oct | 290 | 290 | 290.00 | 3.41 | 0.10 | 1 |
| Bs_Oct | 89 | 89 | 89.00 | 2.46 | 0.15 | 1 |
| C40_Oct | 488 | 488.25 | 488.63 | 4.95 | 0.02 | 0.999914 |
| C53_Oct | 323 | 323.5 | 323.63 | 4.57 | 0.03 | 0.999904 |
| C60_Oct | 368 | 368 | 368.16 | 4.01 | 0.10 | 0.999973 |
| Cs_Oct | 85 | 85 | 85.31 | 2.44 | 0.17 | 0.99997 |
